# Expression study of *cadherin7 *and *cadherin20 *in the embryonic and adult rat central nervous system

**DOI:** 10.1186/1471-213X-8-87

**Published:** 2008-09-19

**Authors:** Masanori Takahashi, Noriko Osumi

**Affiliations:** 1 Division of Developmental Neuroscience, Center for Translational and Advanced Animal Research, Tohoku University Graduate School of Medicine, 2-1, Seiryo-machi, Aoba-ku, Sendai, 980-8575, Japan; 2 The Core Research for Evolutional Science and Technology (CREST), Japan Science and Technology Agency (JST), 4-1-8, Honmachi, Kawaguchi, 332-0012, Japan

## Abstract

**Background:**

Vertebrate classic cadherins are divided into type I and type II subtypes, which are individually expressed in brain subdivisions (e.g., prosomeres, rhombomeres, and progenitor domains) and in specific neuronal circuits in region-specific manners. We reported previously the expression of *cadherin19 *(*cad19*) in Schwann cell precursors. *Cad19 *is a type II classic cadherin closely clustered on a chromosome with *cad7 *and *cad20*. The expression patterns of *cad7 *and *cad20 *have been reported previously in chick embryo but not in the developing and adult central nervous system of mammals. In this study, we identified rat *cad7 *and *cad20 *and analyzed their expression patterns in embryonic and adult rat brains.

**Results:**

Rat cad7 protein showed 92% similarity to chick cad7, while rat cad20 protein had 76% similarity to *Xenopus *F-cadherin. Rat *cad7 *mRNA was initially expressed in the anterior neural plate including presumptive forebrain and midbrain regions, and then accumulated in cells of the dorsal neural tube and in rhombomere boundary cells of the hindbrain. Expression of rat *cad20 *mRNA was specifically localized in the anterior neural region and rhombomere 2 in the early neural plate, and later in longitudinally defined ventral cells of the hindbrain. The expression boundaries of *cad7 *and *cad20 *corresponded to those of region-specific transcription factors such as *Six3*, *Irx3 *and *Otx2 *in the neural plate, and *Dbx2 *and *Gsh1 *in the hindbrain. At later stages, the expression of *cad7 *and *cad20 *disappeared from neuroepithelial cells in the hindbrain, and was almost restricted to postmitotic cells, e.g. somatic motor neurons and precerebellar neurons. These results emphasized the diversity of *cad7 *and *cad20 *expression patterns in different vertebrate species, i.e. birds and rodents.

**Conclusion:**

Taken together, our findings suggest that the expression of *cad7 *and *cad20 *demarcates the compartments,   boundaries, progenitor domains, specific nuclei and specific neural circuits during mammalian brain development.

## Background

In the early neural plate, the brain primodium is subdivided into several domains, i.e., neuromeres, to generate regional differences and units [1]. The hindbrain primordium is morphologically divided into lineage-restricted functional metameric units called rhombomeres [2]. Neuroepithelial cells are initially scattered in the hindbrain neuroepithelium, but are sorted gradually into each compartment [3]. In the forebrain, that consists of the diencephalon and telencephalon, the primordium is longitudinally divided into prosomeres, which are defined by the expression border of various transcription factors such as homeodomain (HD) proteins [4,5], and a lineage restriction of neuroepithelial cells between each subdivision has been reported in the caudal diencephalon of the chick embryo [6]. In each region of the hindbrain and spinal cord, the neuroepithelium is also regionalized into basal and alar plates, which are separated at a groove called the sulcus limitans. Several molecular makers, e.g., HD transcription factors, subdivide the neuroepithelium into several discrete progenitor domains that give rise to different types of neurons along the dorsoventral (D-V) axis [7].

Previous studies demonstrated the expression of cadherin superfamily genes encoding cell adhesion molecules in the brain and the spinal cord, with distinct expression patterns that correspond with the subdivisions of the brain and the spinal cord [8-12]. These studies proposed that cadherin-mediated differential cell affinity establishes various compartments and regionalizes the neuroepithelium. Vertebrate cadherin superfamily genes are categorized into subfamilies, such as classic cadherins, protocadherins, and desmosomal cadherins [13]. Classic cadherins have cadherin-repeats in an extracellular region called EC (extracellular cadherin) domain and associate with β-catenin and p120-catein in the cytoplasmic domains that connect to the actin cytoskeleton [13]. The EC1 domain of classic cadherin shows adhesive properties that enhance the homophilic binding of cadherin. Classic cadherins are categorized into type I and type II groups with or without conserved amino acids, His-Ala-Val (HAV) within EC1 domain [14]. The adhesive affinities of type I cadherins have been studied extensively. Cells expressing single type I cadherin, such as E-cadherin or N-cadherin, prefer to adhere to those expressing the same cadherin via homophilic interaction rather than heterophilic binding. For example, the neuroectoderm is segregated from the ectoderm by distinct affinity of N-cadherin and E-cadherin during the formation of the neural tube [15]. In contrast, R-cadherin and N-cadherin, which are type I cadherins, interact in a heterophilic manner [16], and the adhesive interaction between individual subtypes of type II cadherin is not always homophilic in nature [17]. Therefore, the complexity of homophilic and/or heterophilic interactions between each type II cadherin subtype may be involved in several developmental processes beyond tissue segregation during early embryogenesis.

*F-cadherin *(*F-cad*), a member of type II classic cadherins, is expressed in the area adjacent to the sulcus limitans, the boundary between the basal and alar plates, which restricts positioning of neuroepithelial cells in *Xenopus *embryos [18,19]. In the chick neural tube, *cadherin7 *(*cad7*) is expressed not only in migratory neural crest cells but also in the domain ventral to the sulcus limitans of the neural tube [20-22]. *R-cadherin *(*R-cad*) and *cadherin6 *(*cad6*) are expressed in the primodia of the mouse cerebral cortex and striatum, respectively, and their differential cell affinities mediate segregation at the corticostriatal boundary [23]. Other subtypes of classic cadherins also have multiple functions in the developing and adult brain, such as involvement in neuronal migration and connectivity of specific neuronal circuits, and synaptic formation in the brain. For example, *cad6*, *cad8 *and *cad11 *are observed in specific neural circuits [24,25], and α N-catenin and N-cadherin, which are localized in the pre- and postsynaptic regions, modulate dendritic spine formation in an activity-dependent manner [26]. So far, more than 20 members of type II classic cadherin have been identified. In Schwann cell precursors, we reported previously the expression of rat *cad19 *gene [27], which is a type II subtype clustered with *cad7 *and *cad20 *on chromosome 13. In mice, *cad7 *is expressed only in adult tissues [28], whereas *cad20*, a *F-cad *homologue, is expressed in early neural tissues [28,29]. However, little is known about the expression patterns of *cad7 *and *cad20 *in the developing and adult rodent brains. In this study, we focused on *cad7 *and *cad20 *genes and analyzed their expression patterns in the developing and adult rat brains. The results showed that the expression borders of *cad7 *and *cad20 *corresponded with those of regional compartments and boundaries, which were marked with the expression of region-specific transcription factors. *Cad7 *and *cad20 *were also expressed in neurons of several nuclei that form the cerebellar/precerebellar circuitry in the late embryonic and adult hindbrain. The results suggest the contribution of *cad7 *and *cad20 *in the formation of compartment/boundary and specific neuronal circuitry in the rat hindbrain.

## Results

### Isolation of rat *cad7 *and *cad20*

**Figure 1:**
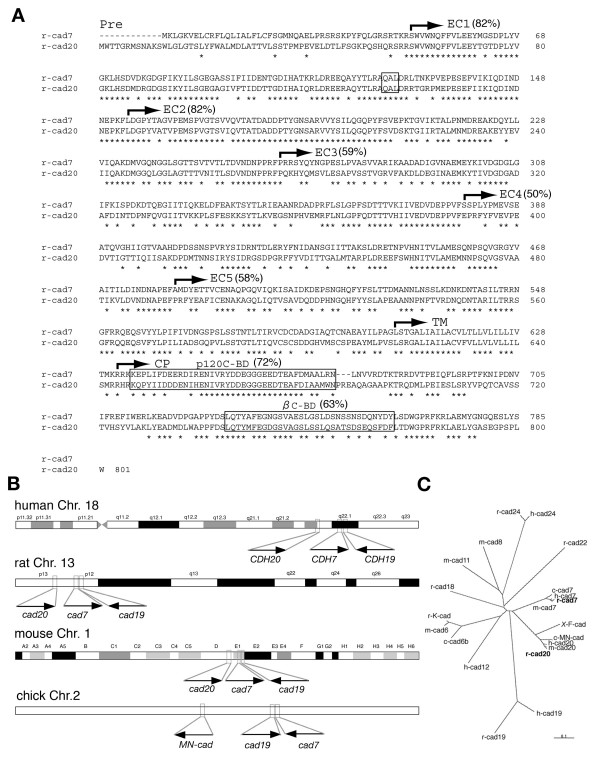
**Amino acid sequences and chromosome mapping of rat *cad7 *and *cad20***. **A**: Alignments of rat cad7 and cad20 protein sequences. Asterisks indicate identical amino acids between cad7 and cad20 proteins. Numbers indicate percentage of identical amino acids in each domain and catenin binding domains. **B**: The positions of *cad7*, *cad19 *and *cad20 *on the human, rat, mouse and chick genomes, based on genome data search. Directions of arrows indicate coding on forward (right) and reverse (left) strands, respectively. **C**: Phylogenetic tree for type II classic cadherins. m, mouse; r, rat; h, human; c, chick; *X*, *Xenopus*.

To examine the expression of rodent homologues of chick *cad7 *and *Xenopus F-cad*, we searched for rat genome sequences orthologous to chick *cad7 *and *Xenopus F-cad*. We found highly conserved sequences on rat chromosome 13p12 and cloned cDNAs covering open reading frames (ORFs) of putative rat cad7 and cad20 proteins by RT-PCR (Fig. 1A, B, Table 1). Classic cadherins consist of EC domains (or cadherin repeats), a transmembrane domain, and cytoplasmic domains. The putative ORF of rat cad7 encoded 785 amino acids and the protein showed 92% similarity with chick cad7 [20], and 96% similarity with human cad7 [17,30] (Fig. 1A). The putative ORF of rat F-cad encoded 801 amino acids, and the protein had 76% similarity with *Xenopus *F-cad [18] (Fig. 1A), 86% similarity with chick MN-cadherin (MN-cad) [31,32] and 95% similarity with human CDH20, a homologue of F-cad [30] (Fig. 1A). β-catenin and p120 catenin binding sites were conserved within the cytoplasmic domains of rat cad7 and cad20 proteins, which showed 72% and 63% identities, respectively (Fig. 1A). The EC1 and EC2 domains of cad7 and cad20 proteins were highly conserved with 82% identities (Fig. 1A). These results indicate that our two-cloned type II classic cadherins are homologues of chick cad7 and *Xenopus *F-cad.

**Table 1:** Characterization of *cad20*/*cad19*/*cad7 *gene cluster in different species.

**Species**	**Gene**	**Chromosome**	**Ensemble Gene model**	**References**
Chick	*MN-cad*	2 (2_19.90)	68,341,649–68,376,741(-)	[31] Price et al., 2002, [32] Shirabe et al., 2005
	*cad19*	2 (2_19.90)	95,067,813–95,101,300(+)	[27] Takahashi and Osumi, 2005, [33] Luo et al., 2007
	*cad7*	2 (2_19.90)	95,357,588–95,418,911(-)	[20,21] Nakagawa and Takeichi, 1995, 1998
Rat	*cad20*	13 (13p12)	10,970,187–11,038,903(+)	In this study
	*cad7*	13 (13p12)	16,884,417–17,020,843(+)	In this study
	*cad19*	13 (13p12)	18,148,715–18,230,353(-)	[27] Takahashi and Osumi, 2005
Mouse	*cad20*	1	104,833,281–104,893,569 (+)	[28] Moore et al., 2004, [29] Faulkner-Jones et al., 1999
	*cad7*	1	109,880,766–110,037,943(+)	[28] Moore et al., 2004
	*cad19*	1	110,788,240–110,854,295(-)	[27] Takahashi and Osumi, 2005
Human	*CDH20*	18 (18q22-q23)	57,308,755–57,373,345(+)	[30] Kools et al., 2000
	*CDH7*	18 (18q22-q23)	61,568,468–61,699,155(+)	[17] Shimoyama et al., 2000, [30] Kools et al., 2000
	*CDH19*	18 (18q22-q23)	62,322,301–62,422,196(-)	[30] Kools et al., 2000

Human *CDH7*, *CDH19 *and *CDH20 *genes are clustered on chromosome18q22-p23 [30]. Accordingly, we examined whether similar gene clusters are conserved in the rat, mouse and chick genomes. *Cad7 *and *cad20 *genes were closely localized in the same chromosome with *cad19 *(Fig. 1B). It is noteworthy that in the chick genome, the position of *cad7*, *MN-cad *and *cad19 *[33] gene cluster on chromosome 2 is different from that of *cad7 *and *cad20 *on human, rat, and mouse genomes (Fig. 1C, Table 1).

### Region-specific expression of *cad7 *in the developing rat embryo

**Figure 2:**
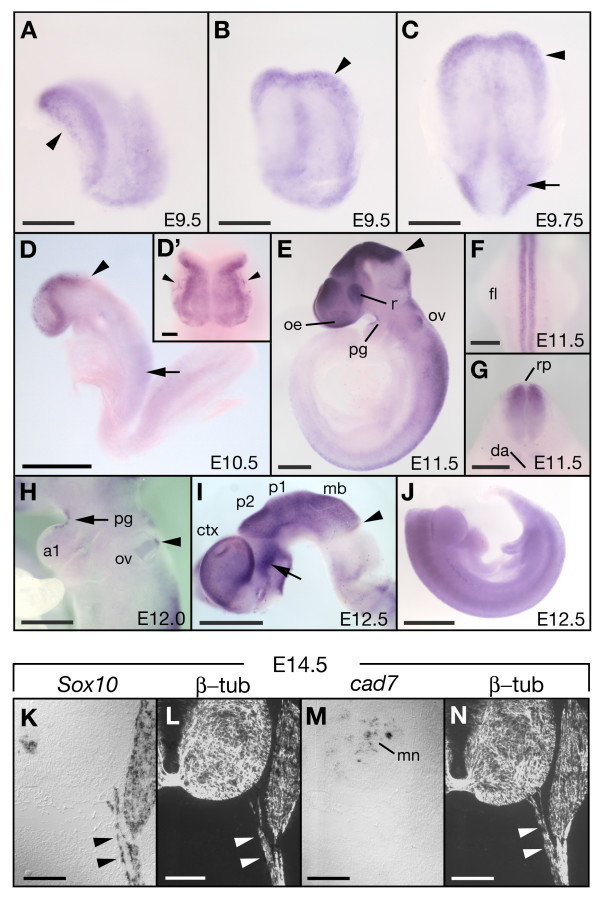
**Expression patterns of *cad7 *in the developing rat embryo**. **A-C**: The expression of *cad7 *mRNA in the anterior margin of the early neural plate at E9.5–9.75 (arrowhead in A, B and C), and in the lateral plate at E9.75 (arrow in C). These pictures show lateral (A), ventral (B) and dorsal (C) views. **D**: Lateral view showing the expression of *cad7 *in the brain region anterior to the midbrain/hindbrain boundary (arrowhead) and caudal neural tube (arrow) at E10.5. **D'**: Dorsal view of D. The expression of *cad7 *is detected at the edge of the neural plate and part of migrating neural crest cells (arrowheads). **E-G**: Expression of *cad7 *in the forebrain and midbrain (E). *Cad7 *is expressed in the dorsal region of the otic vesicle (ov), and in the olfactory epithelium (oe) and retina (r) at E11.5 (E). G is a cross-section at the fore limb (fl) level. In the hindbrain and spinal cord, *cad7 *is expressed in the dorsal neuroepithelium and the expression is absent in the roof plate (rp) (F, G). E and F images indicate lateral and ventral view, respectively. **H**: Lateral view of *cad7 *expression in the pharyngeal region at E12.0. Arrow and arrowhead indicate expression of *cad7 *in the pharyngeal groove (pg) and otic vesicle (ov), respectively. **I-J**: Lateral view of *cad7 *staining in the brain of E12.5 embryos. Arrow in I indicates the expression of *cad7 *in the ventral domains of prosomere3 (p3) and secondary prosencephalon. No expression of *cad7 *in the dorsal root ganglion cells at the trunk level (J). **K-N**: On cross-sections from E14.5 embryo, *cad7 *transcripts are detected in the subpopulation of motor neurons (mn) but not in the dorsal root ganglion or Schwann cell precursors (arrowheads in K, and M) expressing *Sox10*, along motor nerve (arrowheads in L and N). al, pharyngeal arch 1; da, dorsal aorta; ctx, cerebral cortex; mb, midbrain. Scale bars: 200 μm in A-C, G and K-N; 400 μm in D; 100 μm in D'; 500 μm in E and H; 300 μm in F; 1 mm in I and J.

We first examined the expression patterns of *cad7 *in the rat embryos by whole mount *in situ *hybridization. Expression of *cad7 *mRNA appeared at the edge of the anterior neural plate at embryonic day (E) 9.5–9.75 and in the lateral plate at the posterior region (Fig. 2A–C). At E10.5 (12 somites stage), rostral expression of *cad7 *was exclusive to the presumptive forebrain, midbrain and caudal neural tube, and then was strongly identified at the edge of the neural plate, which corresponds to neural crest cells (Fig. 2D, D'). *Cad7 *was expressed in migrating neural crest-derived cells in the cephalic region (Fig. 2D'), but disappeared at later stages. At E11.5, *cad7 *was expressed in the rostral brain regions including the telencephalon, diencephalon and midbrain, and the sharp posterior border of the expression apparently corresponded to the midbrain/hindbrain boundary (MHB) (Fig. 2E). In the caudal hindbrain and the spinal cord, *cad7 *was expressed in the dorsal neuroepithelium but no expression was noted in the roof plate (Fig. 2F, G). *Cad7 *expression also appeared in the olfactory epithelium, retina, dorsal area of the otic vesicle and the pharyngeal groove at E11.5 (Fig. 2E). At E12.0, the pattern of *cad7 *expression was similar to that at E11.5 (Fig. 2H). At E12.5, *cad7 *expression extended to the cerebral cortex, the diencephalon including the presumptive pretectum (p1), thalamus (p2) and prethalamus (p3), and the dorsal midbrain (Fig. 2I). Immunostaining of the chick diencephalon with antibody against cad7 showed that cad7 expression demarcated the pretectum, ventral thalamus and the zona limitans intrathalamica (ZLI) but not the dorsal thalamus [34], suggesting that the *cad7*-expressing diencephalic region is not conserved in ZLI and dorsal thalamus in the rat and chick. Previous studies demonstrated the expression of cad7 in chick neural crest-derived cells including Schwann cells and their precursors [20,21]. In E12.5 rat embryo, *cad7 *mRNA was undetectable in the dorsal root ganglia and neural crest-derived migrating cells at the trunk level (Fig. 2J). We further examined *cad7 *expression in Schwann cell precursors at E14.5 [27]. We performed *in situ *hybridization on serial sections for *cad7 *and *Sox10*, a maker of neural crest-derived cells [35], and immunostaining of the same sections with β-tubulin antibody (Tuj1) to detect the nerve of motor neurons (Fig. 2K–N). In contrast to the expression of *Sox10 *in Schwann cell precursors on the motor nerve, *cad7 *was not expressed in these cells (Fig. 2K and 2M). These results suggest that rat *cad7 *gene is activated in the central but not peripheral nervous system during neural development.

### Expression borders of *cad7 *and *cad20 *are adjacent to those of transcription factors in the early neural plate

**Figure 3:**
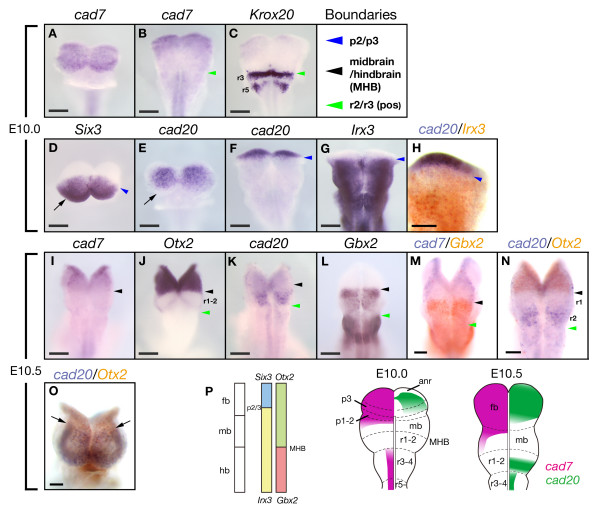
**Expression of *cad7 *and *cad20 *and transcription factors in the early neural plate**. Images shown in A, D, E, O and B, C, F-N are taken from the anterior and dorsal sides, respectively. **A-I**: *Cad7 *mRNA is expressed in the forebrain region (A). The anterior border of *cad7 *expression in the hindbrain is consistent with that of *Krox20 *in the r3 (green arrowhead in B and C). At E10.5, *cad20 *is expressed in the forebrain region and the expression region overlaps with that of *Six3 *(D, E). *Cad20 *expression is absent in the anterior margin of the neural plate (arrow in E), which is different from *Six3 *expression (arrow in D). Blue arrowheads in D and G indicate the expression boundary between *Six3 *and *Irx3*. The boundary between *cad20*- and *Irx3*-domains demarcates the position of the presumptive zona limitans intrathalamica (ZLI) (arrowhead in F, G and H). **I-O**: At E10.5, the posterior border of *cad7 *expression corresponds to the midbrain/hindbrain boundary (MHB) as indicated by *Gbx2 *(black arrowhead in M). The border of *cad20 *expression is not consistent with the posterior border of *Otx2 *(black arrowhead in N), the expression is detected in the r2 region expressing *Gbx2*. The posterior border of *cad20 *is detected anterior to forebrain/midbrain boundary (arrows in O). **P**: Schematic illustrations of mapping of *cad7 *and *cad20 *in the early stages. pos, pre otic sulcus; fb, forebrain; mb, midbrain; hb, hindbrain, anr, anterior neural ridge; MHB, midbrain/hindbrain boundary. Scale bars: 200 μm in A-H; 400 μm in I-L; 200 μm in M-O.

Since previous reports suggested cadherins expression in specific regions corresponding to prosomere and rhombomere compartments [36,37], we next examined the expression borders of *cad7*, *cad20 *and compared their expressions with those of transcription factors showing region-specific expression patterns at early development stages. At E10.0 (6-somites stage), *cad7 *expression became restricted to the anterior region including the forebrain and caudal hindbrain (Fig. 3A). The anterior border of *cad7 *in the hindbrain corresponded to that of *Krox20 *(Fig. 3B, C), a zinc-finger transcription factor, at rhombomere 3 (r3) [38]. The expression of *cad20 *was more restricted in the anterior region and overlapped with the expression of *Six3 *[39], a HD protein (Fig. 3D, E). A previous report showed that the prospective position of the zona limitans intrathalamica (ZLI) is demarcated by the expression boundaries of *Six3 *and *Irx3*, HD transcription factors, in the chick embryo [40]. Since the posterior border of *cad20 *expression was adjacent to the anterior border of *Irx3 *(Fig. 3F–H), *cad20 *expression indicates the prospective position of ZLI in the rat neural plate.

Next, we compared the expression domains of *cad7 *and *cad20 *with those of transcription factors at E10.5 stage (10–12 somite stage). *Otx2 *and *Gbx2 *are HD transcription factors that establish the MHB with mutual repression [41]. Remarkably, the posterior border of *cad7 *expression corresponded with the MHB (Fig. 3I, M). The second domain of *cad20 *was identified in the hindbrain in r2 region at areas positive for *Gbx2 *(Fig. 3K, L and 3N) whilst the expression of *Otx2 *was excluded (Fig. 3J, N). At E10.5, the posterior border of *cad20 *in the forebrain became sharper (Fig. 3O). These results indicate that the expression borders of *cad7 *and *cad20 *correspond to the prosomere and rhombomere boundaries in the early neural plate.

### *Cad7 *and *cad20*-expressing domains correspond to progenitor domain boundary along the D-V axis

**Figure 4:**
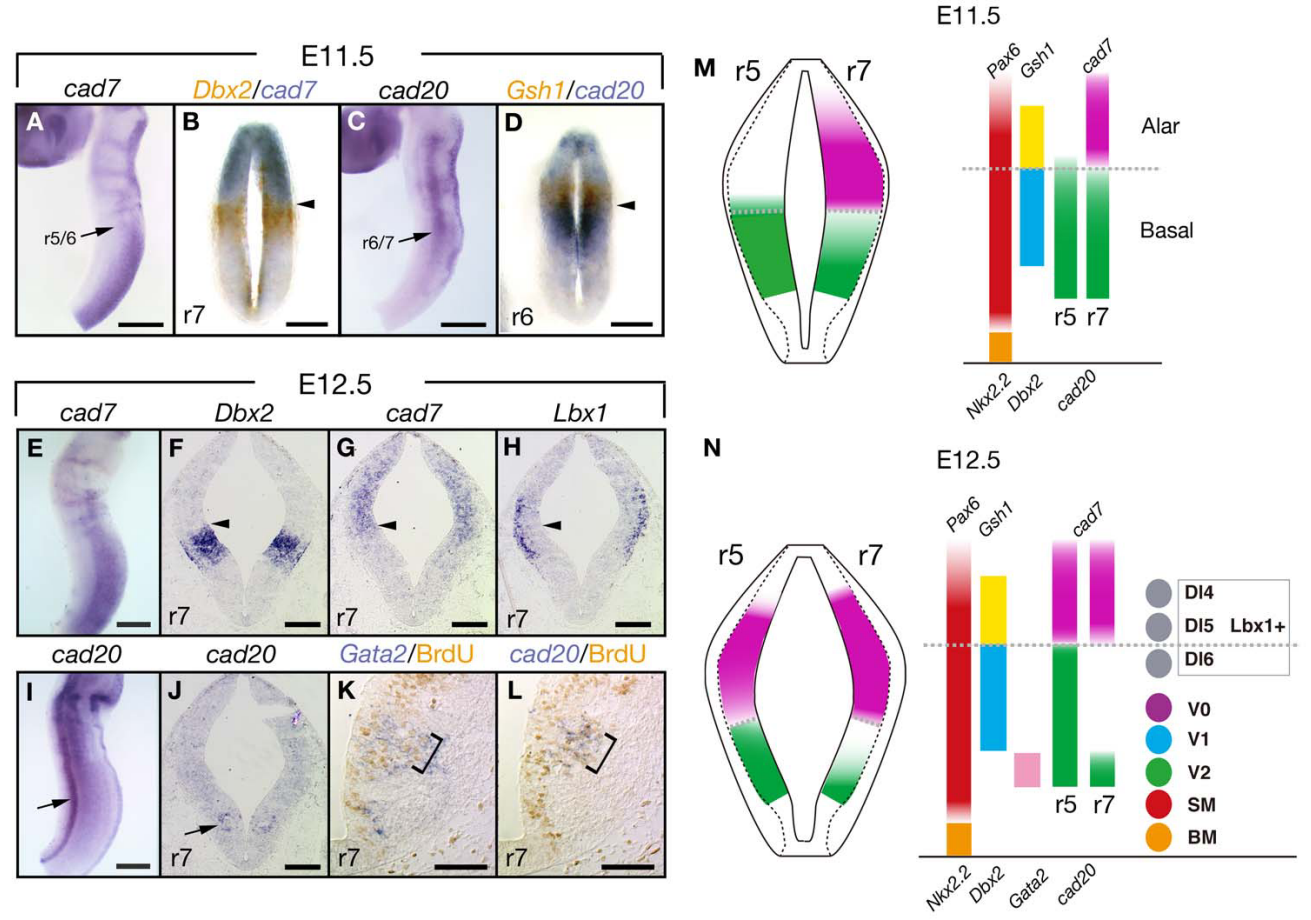
**Mapping of *cad7 *and *cad20 *expression on progenitor domains in the hindbrain**. **A-D**: Expression analysis on the dissected whole brain (A, C) and cross-sections at r7 (B) and r6 level (D) of E11.5 embryo. Images of A and C are taken from the lateral side of the brain. At E11.5, *cad7 *mRNA is mainly detected in the dorsal domain of the neural tube posterior to r5/6 boundary (arrow in A), and the ventral border of *cad7 *corresponds to the dorsal border of *Dbx2 *(arrowhead in B, cross-section at r7 level after double detection). At E11.5, *cad20 *transcripts are highly detected in the middle domain of the hindbrain anterior to r6/7 boundary (arrow in C), and the dorsal area of *cad20*-domain overlaps with the ventral area of *Gsh1*-domain (arrowhead in D, cross-section at r6 level after double detection). **E-J**: Expression analysis on the dissected whole brain (E, I) and serial cross-sections at r7 level (F-H, and J) from E12.5 embryo. Images of E and I are taken from the lateral side of the brain. The ventral border of *cad7 *in the dorsal progenitor domain is consistent with the dorsal border of *Dbx2 *(arrowheads in F and G), and part of *cad7*-expressing progenitors gives rise to *Lbx1*-positive dorsal interneurons (Dl4-6) (H). The expression of *cad20 *in the middle domain of the hindbrain gradually disappears at E12.5 (I), and another expression domain of *cad20 *also appears in a more ventral region (arrow in I and J). **K-L**: BrdU detection after *in situ *hybridization. The domain of *cad20*-expressing cells corresponds to that of cells expressing *Gata2*, a V2 interneuron lineage maker (brackets in K and L), and *cad20 *expressing cells are progenitor cells incorporating BrdU (arrow in L). **M-N**: Summary of expression of *cad7 *and *cad20 *along D-V axis at E11.5 (M) and E12.5 (N). Left: expression domains at r5 and r7. Right: progenitor domains defined with expressions of homeodomain transcription factors and cadherins. Broken lines indicate the border between basal and alar plates. V0–V2, V0–V2 interneuron; SM, somatic motor neuron, BM, branchial motor neuron. Scale bars: 500 μm in A, C, E and I; 150 μm in B, D; 200 μm in F, G, H and J; 200 μm in K, L.

The hindbrain and spinal cord are subdivided into the basal plate and alar plate, which give rise to both motor/interneurons and interneurons, respectively. The boundary between these plates (known as the sulcus limitans) is visualized as a groove at the apical surface of the neural tube [42]. Previous reports suggested that the dorsal borders of *Xenopus F-cad *and chick *cad7 *correspond with the position of the sulcus limitans as defined by HD transcription factors [18,19,22], suggesting that the basal/alar boundary can be detected by differential expression of type II cadherin subtypes as molecular makers. Therefore, we investigated whether rat *cad7 *and *cad20 *mark the alar/basal boundary and discrete progenitor domains in the developing hindbrain. At E11.5 prior to differentiation of interneurons [43], *cad7 *mRNA was expressed in dorsal progenitor cells at r6-7 in the hindbrain (Fig. 4A), and *cad20 *was longitudinally expressed in the midline of the hindbrain neuroepithelium at r1-6 (Fig. 4C). To examine the correlation between the expression domain and progenitor domains, which are subdivided by the different expression patterns of HD proteins [7,43], we performed *in situ *hybridization using probes for *Gsh1 *and *Dbx2 *[44,45]. Double staining for *cad7 *and *Dbx2 *in E11.5 embryos indicated that the ventral border of *cad7 *was adjacent to the dorsal border of *Dbx2*-domain (Fig. 4B). Although *cad20 *was somewhat expressed in the domain ventral to *Gsh1*-domain, the dorsal border of *cad20 *overlapped with the *Gsh1*-domain (Fig. 4D).

Even at E12.5, the expression domain of *cad7 *was clearly maintained in neuroepithelial cells in the dorsal hindbrain (Fig. 4E). The ventral border of *cad7 *coincided with the dorsal border of *Dbx2*, and the *cad7 *domain partially overlapped with the progenitor domains of the dorsal *Lbx1*-expressing interneurons [46] (Fig. 4F–H). Remarkably, a narrow longitudinal stripe of *cad20 *expression appeared in the ventral region of the hindbrain (Fig. 4I, J). This pattern was similar to the ventral expression of *cad20 *in the mouse embryo [28]. To elucidate the relationship between the expression domain of *cad20 *and the neural progenitor domain, we compared the expression of *cad20 *with several progenitor domain markers. The results showed similar expression patterns for transcription factor *Gata2 *in *cad20 *domain and the V2 interneuron progenitor domain (Fig. 4K, L) [47]. *Cad20 *expression was also detected in BrdU-incorporated S-phase cells (Fig. 4L). Taken together, these results suggest that *cad7 *and *cad20 *are expressed in different progenitor cells in the developing hindbrain.

### *Cad7 *expression delineates rhombomere boundaries

**Figure 5:**
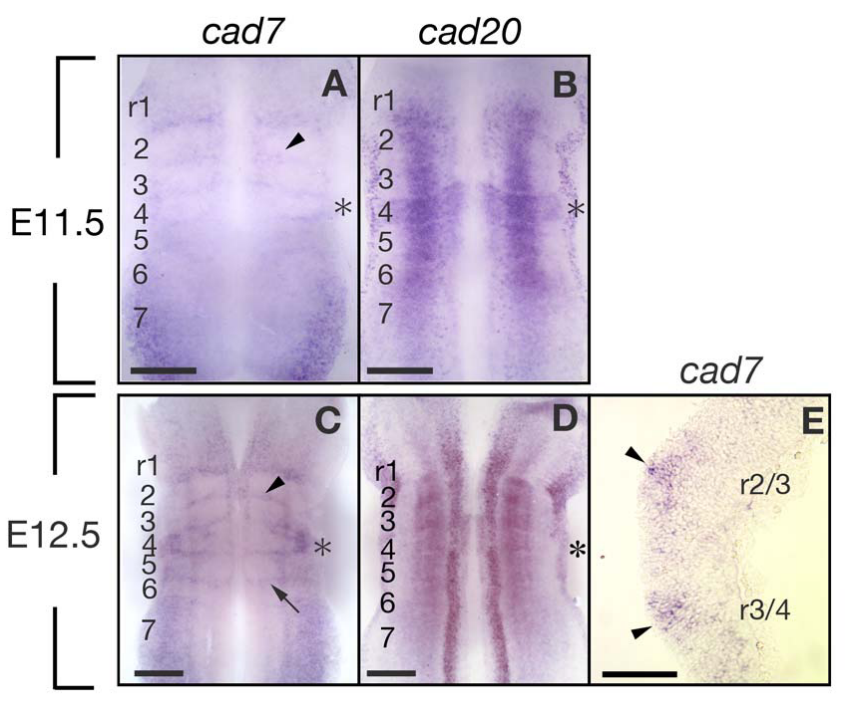
**Expression of *cad7 *and *cad20 *in the rhombomeres and boundaries**. **A-D**: Expression of *cad7 *and *cad20 *in E11.5 (A, B) and E12.5 (C, D) hindbrains which are prepared in open-book style. *Cad7 *transcripts are localized in the boundaries (arrow and arrowhead in A, C). *Cad20 *is strongly expressed in the middle area in the r1-6 and r4 (asterisk in B). **E**: Horizontal section of whole-mount staining embryos with *cad7 *probe. *Cad7 *mRNA is highly expressed in the rhombomere boundary cells (arrowheads in E). Scale bars: 500 μm in A-D; 200 μm in E.

In the hindbrain, cells at the interface between each rhombomere are specialized as rhombomere boundary cells. Interestingly, the expression of *cad7 *was observed in the rhombomere boundary regions (Fig. 5A), while *cad20 *was continuously expressed along the r1–r7 with low level in r7 at E11.5 (Fig. 5B). In the caudal hindbrain, the expression of *cad7 *was downregulated in the ventral domain (Fig. 5A). In addition to longitudinal expression in the middle domain throughout the r1-7, *cad20 *specifically marked r4 at E11.5 (Fig. 5B). At E12.5, the *cad7 *expression was restricted to the rhombomere boundaries and to the dorsal region of the hindbrain (Fig. 5C). The transcripts of *cad7 *were accumulated in the cell body of boundary cells that are located in the apical side (Fig. 5E). The expression of *cad20 *was restricted in the ventral domains of the hindbrain (Fig. 5D). These results suggest possible distinct affinity of neuroepithelial cells mediated by *cad7 *and *cad20 *in the developing rat hindbrain along the A-P axis.

### *Cad7 *and *cad20 *expression in motor neurons and precerebellar neurons at later developmental stages

**Figure 6:**
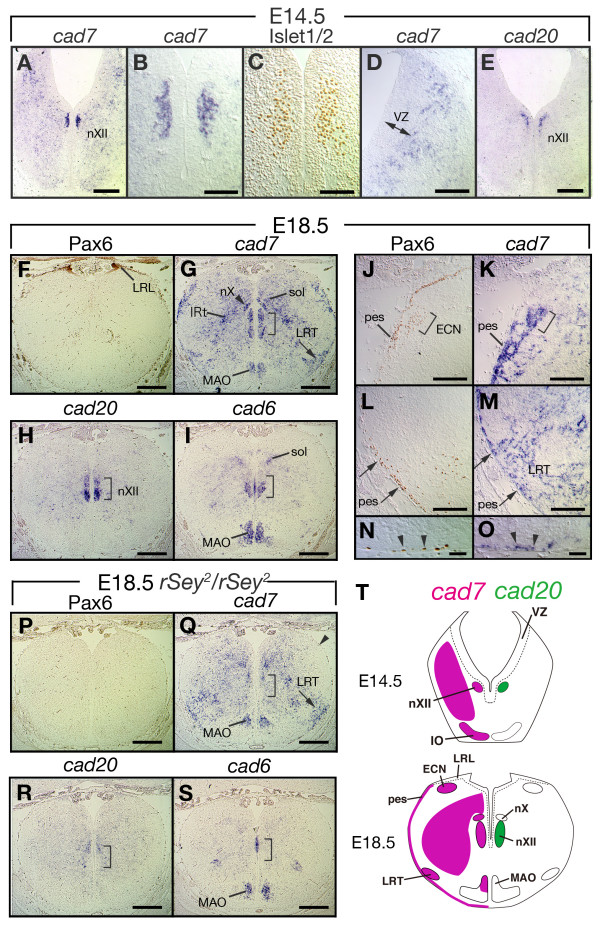
**Expression of *cad7 *in the motor neurons and precerebellar systems**. **A-E**: Expression of *cad7 *and *cad20 *on serial cross-sections from the hindbrain of E14.5 rat embryo at r7 level. (B) High magnification of the image shown in A. Expression of *cad7 *and *cad20 *is detected in hypoglossal motor nuclei (nXII) expressing Islet1/2 (A, B, C and E). No expression of *cad7 *is seen in the ventricular zone (VZ) of the dorsal area (D). **F-O**: Expression of Pax6, *cad7 *and *cad20 *on serial cross-sections at E18.5. Pax6 protein is detected in both the lower rhombic lip (LRL) (F), posterior extramural migrating stream (pes) (J and arrows in L) and the external cuneate nucleus (ECN) (bracket in J). *Cad7 *is expressed in nXII (bracket in G), vagus motor nuclei (nX) (arrowhead in G), lateral reticular nuclei (LRT) (arrow in G), the ECN (bracket in K), the nucleus of the solitary tract (Sol), and in the intermediate reticular zone (IRt). *Cad7*-expressing cells are migrating on the surface of the brain, which is similar to Pax6-expressing cells (arrows in L, M and arrowheads in N and O). *Cad7 *is also expressed in the medial accessory olive (MAO) nuclei expressing *cad6 *(G and I). *Cad20 *is expressed in nXII but not in nX (bracket in H). **P-S**: Serial cross-sections of E18.5 *Pax6 *homozygous mutant rat (*rSey*^2^/*rSey*^2^). Pax6 protein is undetectable (P), while expression of *cad7 *is detected in LRT (arrow in Q) and MAO expressing *cad6 *(S). nXII motor nuclei and ECN are missing in the *Pax6 *homozygous mutant (bracket and arrowhead in Q, respectively). The expression of *cad20 *is also undetectable, which is similar to the *cad6 *expression (bracket in R and S). **T**: Summary of expression of *cad7 *and *cad20*. Scale bars: 300 μm in A and E and F-I; 100 μm in B and C; 200 μm in D, J-M and P-S; 50 μm in N and O.

Previous studies of mouse and chick embryos showed the expression of subtypes of type I and type II classic cadherins in motor neurons and precerebellar neurons, and the involvement of such expression in the formation of each motor pool and in cell migration [24,25,31,48]. We examined the expression of *cad7 *and *cad20 *at later developmental stages in the hindbrain. At E14.5 in the caudal hindbrain, the expression of *cad7 *was downregulated in the ventricular zone but became detectable in the marginal zone (Fig. 6A, D). Judging from the expression of Islet1/2 in motor neurons, *cad7 *and *cad20 *were apparently expressed in the hypoglossal somatic motor nuclei (nXII) (Fig. 6B, C, E). At E18.5, *cad7 *expression extended beyond the nXII into the vagus motor nuclei (nX) (Fig. 6G). *Cad20 *was still expressed in the nXII at E18.5 (Fig. 6H). *Cad7 *was also expressed in the dorsal region including the nucleus of solitary tract (Sol) and in the intermediate reticular zone (IRt) (Fig. 6G).

**Figure 7:**
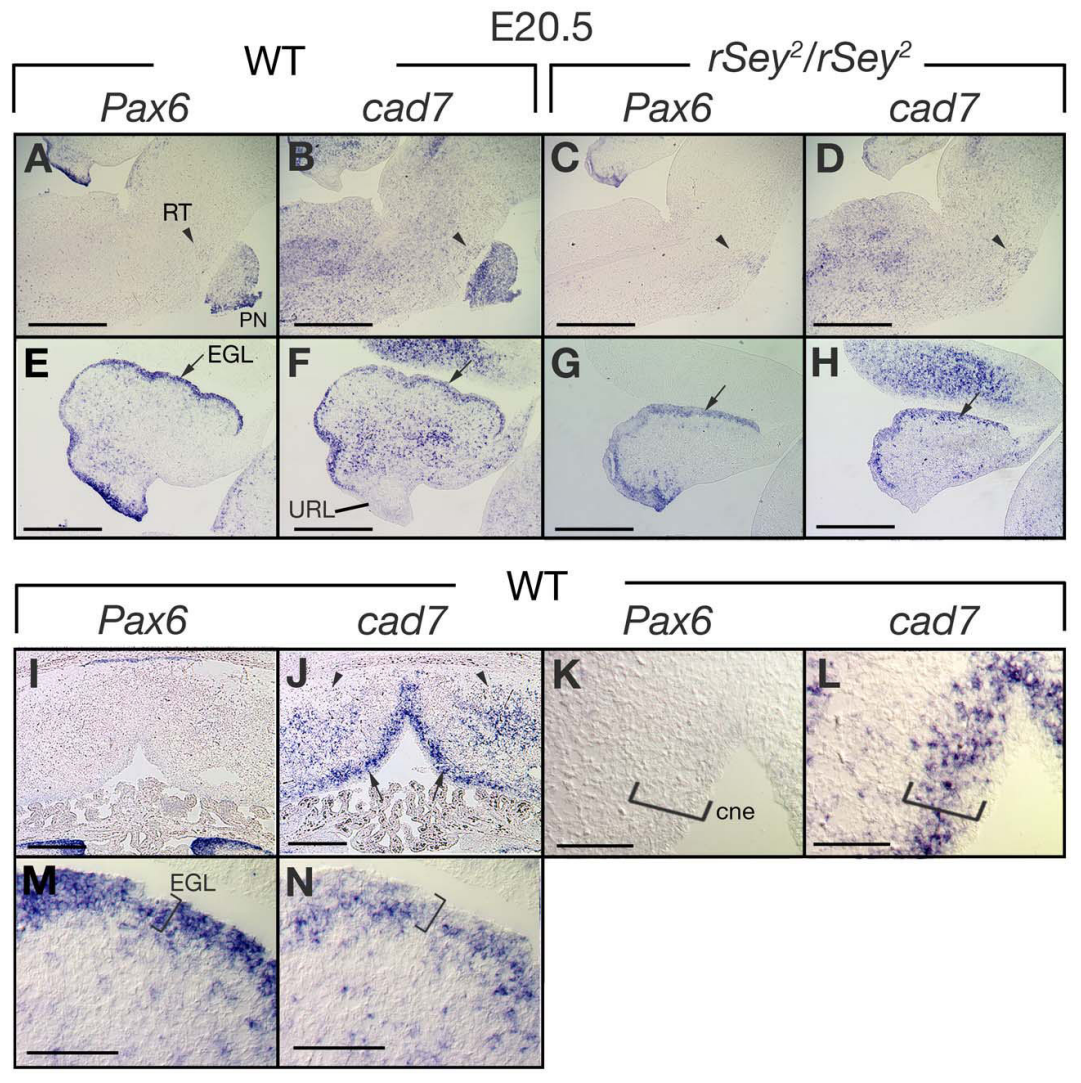
**Expression of *cad7 *in the brainstem and cerebellum in the foetus**. **A-J**: Comparison of expression patterns of *Pax6 *and *cad7 *in the brainstem and cerebellum of the wild type (A, B, E and F) and *Pax6 *homozygous mutant rat (*rSey*^2^/*rSey*^2^) (C, D, G and H) on serial sagittal sections. At E20.5, *Pax6 *and *cad7 *are expressed in the pontine nucleus (PN), reticular nucleus (RT) (arrowheads in A and B) and the external germinal layer (EGL) (arrows in E and F). In the *Pax6 *mutant, *cad7 *expression is detected in the remaining RT (arrowhead in D), which is similar to *Pax6 *expression (arrowhead in C). The expression of *Pax6 *and *cad7 *is observed in EGL of the *Pax6 *mutant (arrows in G, H). **I-L**: Cross-sections of the E20.5 rat cerebellum. *Cad7 *is also expressed in the cerebellar neuroepithelium (cne) (arrows in J, bracket in L) and cerebellar deep nuclei (arrowheads in J) in contrast to the expression of Pax6 (I and K). **M-N**: Higher magnifications of E and F. The expression of *Pax6 *and *cad7 *is detected in both EGL (bracket) and migrating cells. URL, upper rhombic lip. Scale bars: 500 μm in A-D; 100 μm in E-H; 300 μm in I and J; 200 μm in K-M.

In the caudal hindbrain, the migratory stream to form precerebellar nuclei, called the posterior extramural migrating stream (pes), is visualized by the expression of certain makers including HD transcription factors such as Pax6 [49]. Pax6 is essential for the development of the cerebellum and formation of precerebellar nuclei [49-51]. At E18.5, Pax6 was expressed in the external cuneate nucleus (ECN) and the lateral reticular nucleus (LRT) (Fig. 6F, J, L). Interestingly, *cad7 *transcripts were also detected in the pes, ECN and LRT but not in the lower rhombic lip (LRL) (Fig. 6G, K, M, O). *Cad7 *mRNA expression was also detected in the *cad6*-expressing medial accessory olive (MAO) [25] but not in other parts of the inferior olive nucleus (IO) (Fig. 6I and 6G), and in the pontine nucleus (PN) and the reticular nucleus (RT) (Fig. 6A, B and Fig. 7A, B). We demonstrated previously that XII motor nuclei, ECN and PN were missing in the *Pax6 *homozygous mutant rat (*rSey*^2^/*rSey*^2^) [51,52]. In fact, the expression of *cad7 *was not detected in normal positions of the nXII, ECN or PN of *rSey*^2^/*rSey*^2 ^embryo, although the expression was normally detected in LRT and MAO (Fig. 6P, Q, S and Fig. 7D). Furthermore, *cad20 *expression in the normal position of the nXII was not detected in *rSey*^2^/*rSey*^2 ^embryo (Fig. 6R).

### *Cad7 *expression in the external germinal layer of the cerebellum

Next, we examined the expression of *cad7 *in the developing cerebellum. At E20.5, *cad7 *expression overlapped with that of *Pax6 *in the external germinal layer (EGL) containing progenitors of granule cells that later migrate inwards from EGL (Fig. 7A, B, E, F, M, N). On the other hand, *cad7 *expression was not detected in the upper rhombic lip (URL) (Fig. 7F), but was identified in the deep cerebellar nucleus and the epithelium of the anterior hindbrain (Fig. 7J and 7L), which differed from the expression of *Pax6 *(Fig. 7I and 7K). Previous studies in the chick cerebellum at later embryonic stages have shown that *cad7 *transcripts are restricted to the Purkinje cell layer and internal granular layer (IGL) and are not in the EGL [53], and that the immunoreactivity of cad7 protein is absent in the ventricular zone of the developing cerebellum [54]. Considered together, the results suggest diversity of *cad7 *expression patterns in the chick and rat cerebellum.

It is has been reported that Pax6 regulates cell adhesion in the cerebral cortex [55,56] and cerebellar granule cell precursors [57]. *R-cadherin *is a downstream gene of *Pax6 *in the ventricular zone of the developing cerebral cortex [55]. *Cad7 *expression was still detected in EGL cells of the *Pax6 *homozygous mutant rat (Fig. 7D and 7H). These results suggest that *cad7 *expression in EGL is regulated by a *Pax6*-independent pathway.

### *Cad7 *and *cad20 *expression in the adult brainstem and cerebellum

**Figure 8:**
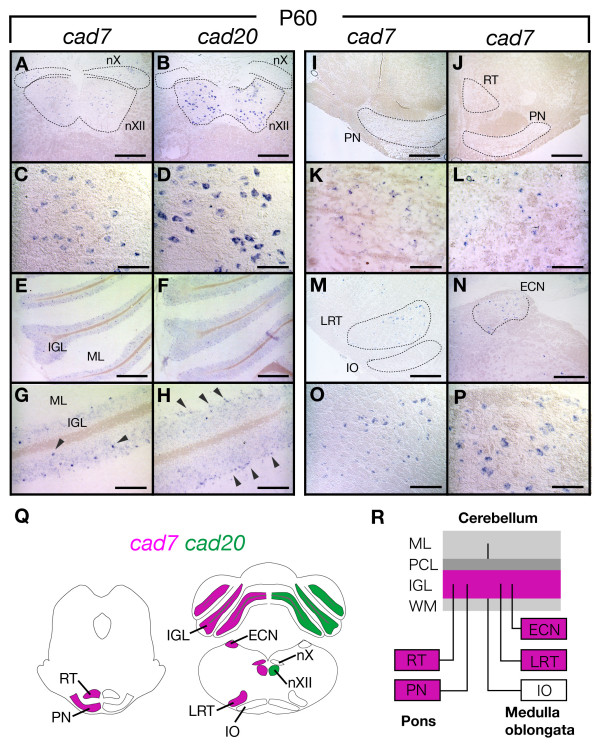
**Expression of *cad7 *in the brainstem and cerebellum in the adult**. **A-H**: Expression of *cad7 *and *cad20 *in motor neurons and cerebellum on cross-sections of 8-week-old (P60) rat. The expression of *cad7 *is detected in both nX and nXII (A) but the expression of *cad20 *is only detected in nXII (B). C and D are high magnification images of *cad7 *and *cad20 *expression in nXII, which are indicated in A and B, respectively. In the cerebellum, *cad7 *and *cad20 *are expressed in the internal granular layer (IGL) (E, F), and scattered large cells expressing *cad7 *are detected in IGL (arrowheads in G, high magnification of E). Intense signal of *cad20 *is observed at the interface between molecular layer (ML) and IGL (arrowheads in H, high magnification of F). **I-P**: Expression of *cad7 *in the pons and medulla oblongata on cross-sections of the P60 rat. Expression of *cad7 *is detected in the pontine nucleus (PN) (I) and reticular nucleus (RT) (J). K and L are high magnification images of *cad7 *expression in the PN and RT indicated in I and J, respectively. *Cad7 *is expressed in the lateral reticular nuclei (LRT) and external cuneate nuclei (ECN) but not in the inferior olive nucleus (IO). O and P are high magnification of *cad7*-expressing cells in the LRT and ECN shown in M and N. **Q-R**: Summary of expression of *cad7 *and *cad20 *(Q) and schematic illustration showing the expression of *cad7 *and neuronal circuits in the hindbrain (R). Lines in R indicate connection between precerebellar neurons and layers of the cerebellum. PCL, Purkinje cell layer; WM, white matter. Scale bars: 500 μm in A, B, E, F, I, J; 400 μm in C, D, K, L, P; 200 μm in G, H, O; 500 μm in E, F, M, N.

In the adult brain, the precerebellar neurons and cerebellar granule cells make synaptic connections. Since *cad7 *was expressed in both precerebellar neurons and progenitors for granule cells, we examined its expression in these neuronal cells in the brainstem and cerebellum of the adult rat (Fig. 8). *Cad7 *was expressed in IGL, which contains granule cells, but not in Purkinje cells (Fig. 8E and 8G). We also observed scattered cells expressing *cad7 *at high levels in IGL (Fig. 8G). *Cad7 *was also expressed in PN and RT, ECN and LRT (Fig. 8I–P) but not in IO (Fig. 8M). On the other hand, *cad20 *was expressed in the IGL but not in the precerebellar nucleus (Fig. 8F, H). The expression of *cad7 *and *cad20 *in nX and nXII was maintained in adulthood (Fig. 8A and 8B). These results suggest that the expression of *cad7 *demarcates the precerebellar/cerebellar system in both embryonic and adult brain.

## Discussion

### Expression of *cad7 *and *cad20 *in early brain subdivision

Wizenmann and Lumsden analyzed rhombomere cells of the chick embryo by re-aggregation assay and were the first to report that segregation of rhombomere neuroepithelial cells between even and odd rhombomeres was probably mediated by calcium-dependent molecules such as cadherins [58]. However, the candidate cadherin molecules expressed in specific rhombomeres or rhombomere boundary cells have not yet been identified in the chick embryo. On the other hand, other studies reported *R-cad *mRNA expression in the midbrain and odd number of rhombomeres, as well as *cad6 *expression in even number of rhombomeres in the mouse neural plate [36,37]. Interestingly, in our analysis, we found specific expression of *cad20 *in the r2 of the rat embryo at E10.5 (Fig. 3) and that the posterior limit of *cad7 *expression was consistent with that of *Otx2 *at E10.5 (Fig. 3). Taking into consideration the transient expression of *R-cad *and *cad6 *at early stages and distinct cell adhesiveness of different cadherins, it is conceivable that classic cadherin subtypes including *cad20 *are involved in segregation of cells at the interface between rhombomeres in rodent embryos. Taken together, it is likely that distinct cadherin subtypes mediate compartmentalization, although further studies are needed to confirm this conclusion.

Lineage restriction of neuroepithelial cells in diencephalic subdivisions has been elucidated in the chick embryo [6,59]. The chick diencephalon is firstly subdivided into three regions; the presumptive pretectum, dorsal thalamus, and ventral thalamus. Previous studies suggested that differential expression of types I and II cadherins demarcates diencephalic subdivisions in the chick and mouse embryos [11,12,34,60]. In the present study, the expression of *cad7 *overlapped with that of *cad20 *in the rat neural plate in the forebrain region. Furthermore, a previous cell lineage analysis suggested that the midbrain/diencephalon boundary restricts cell mixing [61,62], and that *cad6 *delineates the midbrain/diencephalon boundary in the mouse neural plate [62]. In fact, the posterior border of *cad7 *became clearer at E11.5 compared with at E10.5 and the sharp expression boundary was maintained at E12.5 as in the case of *Otx2 *expression (Fig. 2E, I). Otx2 regulates the cell adhesion property of neuroepithelial cells in mice [37], and overexpression of *Otx *can induce cell aggregation in zebrafish embryos [63]. ZLI is not a cell population derived from specialized cells at p2/3 boundary, but is itself a compartment that originates from the area that does not express *Lunatic fringe *(*L-fng*) [64]. The p2/3 border is demarcated by the expression of *Six3 *and *Irx3 *in the early neural plate [40], where ZLI is established. However, in the early neural plate, whether the p2/3 boundary restricts cell mixing has not been elucidated in both avian and rodent embryos. Interestingly, we found that the posterior border of *cad20 *expression was consistent with the p2/3 border, which is mediated by mutual repression of *Six3*/*Fez1/Fez like1 *and *Irx3/Irx1 *(Fig. 3) [40,65]. Our finding suggests the involvement of *cad20 *restricted expression in establishment of ZLI-signalling centre formed at the p2/3 boundary.

### Expression of *cad7 *and *cad20 *in the hindbrain progenitor domains and rhombomere boundary

Although the hindbrain and spinal cord are subdivided into the basal and alar plates at the sulcus limitans defined by a morphological groove in the vertebrates, a recent study using molecular markers has shown that the basal/alar boundary corresponds to the dorsal border of cad7 expression in the chick neural tube [22]. Our study showed that longitudinal expression of *cad20 *resembles those of chick cad7 and *Xenopus F-cad *at the hindbrain level (Fig. 4) [18,20] and that the sulcus limitans in the rat hindbrain is somewhat related to the expression of *cad20*. Remarkably, along the anterior-posterior (A-P) axis, *cad20 *mRNA expression was gradually downregulated in the basal plate from r7 to the spinal cord, and restricted in V2 interneuron progenitor cells (Fig. 5). Moreover, the *cad7*-expressing domain continued through the caudal hindbrain to the spinal cord (Figs. 2, 4 and 5), suggesting that the basal/alar boundary could be established or maintained by distinct cadherin subtypes in the rostral hindbrain and spinal cord. It was noteworthy that both *cad7 *and *cad20 *were expressed at r6 in E11.5 along the D-V axis (Fig. 4), and double staining for *Dbx2*/*cad7 *and *Gsh1*/*cad20 *showed partial overlap of *cad7 *and *cad20 *expression at r6 level in the E11.5 hindbrain (Fig. 4). Since heterophilic adhesion has been reported within distinct type II cadherins [17], and EC1, which is a critical sequence for calcium-dependent adhesion, is highly conserved between cad7 and cad20 proteins (Fig. 1), determination of the adhesion properties of cad7 and cad20 proteins should be the next research priority.

In the ventral spinal cord and hindbrain, transcription factors such as Pax, Nkx and Dbx families regulate the formation of progenitor domains by HD codes and specification of neuronal subtypes by mutual repression activities [7,43]. In the chick spinal cord, ectopically expressed Pax7 can repress cad7 in the ventral region and Shh can induce cad7 in the dorsal region [66]. Therefore, it is possible that rat *cad7 *and *cad20 *are regulated by Shh signalling pathway.

A previous study indicated that segregation of boundary cells is mediated by radical fringe-mediated activation of the Notch signalling pathway in the zebrafish hindbrain [67]. In the mouse, although three fringe genes are not expressed at rhombomere boundaries [68], expression of Hes1, a target gene of Notch signalling, persists at high levels in boundary cells in the hindbrain [69]. Rhombomere boundary cells exhibit a static feature contrary to rhombomere centre cells, i.e., a slow rate of proliferation and interkinetic nuclear migration, and their nuclei are located on the ventricular surface [70]. Since *cad7 *expression in rhombomere boundaries actually starts after the formation of boundaries, such expression at rhombomere boundaries implicates differential cell adhesiveness between boundary and non-boundary cells in maintenance of boundary cells. Considering the expression of cad7 in ZLI, a boundary in the chick diencephalon [34], our findings could be interpreted to mean that cadherin-mediated cell-to-cell contact serves to restrict intermingling of boundary cells and compartment cells, thereby maintaining boundary regions.

### Expression of *cad7 *in precerebellar neurons and cerebellum during late embryonic and adult periods

Interestingly, the expression of *cad7 *and *cad20 *mRNAs in the neuroepithelium disappeared by E14.5, and their expression was detected in postmitotic neurons in the pons and medulla oblongata (Figs. 6 and 7). Previous studies reported that cadherins also contribute to cell migration in the brain [48]. In the cerebellum, Purkinje cells are generated from epithelial progenitor cells and migrate to the deep layer [71]. Experimentally, *cad7 *and *cad6b*-overexpressed progenitor cells preferentially migrated into Purkinje cell domain, which endogenously expresses these cadherins in the chick cerebellum [72]. The lower RL cells generate four types of precerebellar neurons to form the pontine, reticulotegmental, lateral reticular and external cuneate nuclei [73,74]. These precerebellar neurons project to the granule cells in the cerebellum [71]. The dorsal cells of the caudal hindbrain generate the inferior olive neurons, and express cadherin subtypes, e.g., *cad6*, *cad8 *and *cad11 *[23,24]. Interestingly, at later stages, we observed switching of *cad7 *expression from epithelial cells to migrating postmitotic neurons. Although the expression of various cadherin subtypes in precerebellar neurons had been reported previously [48], the expression of *cad7 *was more specifically detected than that of other cadherins in all precerebellar nuclei. Neurons expressing *cad7 *(X/XII cranial motor neurons, pontine and external cuneate neurons) aggregated at late embryonic stages (Figs. 6 and 7), suggesting the involvement of *cad7 *in cell sorting mechanisms, in agreement with the functions of type II classic cadherin subtypes in the chick spinal cord [31].

We found sparse *cad7 *expression in precerebellar neurons in the adult hindbrain (Fig. 8). The cadherin-catenin complex including N-cadherin/αN-catenin is involved in the formation of synaptic contact [26,75]. Furthermore, recent studies have shown that type II subtypes, *cad11 *and *cad13*, also have specific roles in synaptic function including modulation of long-term potential and neurotransmission [76,77], and that *cad8 *has an important role in transmission of sensory information from sensory neurons to the dorsal horn neurons in the spinal cord [78]. Therefore, the expression pattern of *cad7 *in the hindbrain and cerebellum suggests that *cad7 *may physiologically modulate the cerebellar/precerebellar neural circuitry.

### Genomic organization of *cad20/cad19/cad7 *cluster and expression among different species

Cadherin family genes evolutionally duplicated [79] and formed as several clusters on chromosomes [80,81]. In the human genome, *CDH5 *(*VE-cad*)/*CDH1 *(*E-cad*)/*CDH3 *(*P-cad*) and *CDH8*/*CDH11*/*CDH13*/*CDH15 *are located on chromosome 16q21/22 [80], and *CDH20*/*CDH19*/*CDH7 *are clustered on 18q22-23 [30]. Comparison of the expression of such clustered cadherin genes among distinct species is important in order to identify common control element. However, our results showed that the distribution of rat *cad7 *and *cad19 *to that of *cad20 *was inconsistent with the localization of their homologues in the chick (Fig. 1, Table 1). Chick *cad7 *is also expressed in neural crest-derived cells as well as in the ventral neural tube [20,21]. Our previous and present studies showed that *cad19 *but not *cad7 *was expressed in the neural crest cell lineage at the trunk level in the rat [27], suggesting that the distribution of *cad7 *and *cad19 *genes is associated with their expression patterns. However, the expression of chick *cad19 *was detected in part of neural crest-derived cells and neural tube but not in the dorsal domain and rhombomere boundary cells in the hindbrain (our unpublished observation). Taken together, our analysis suggests that the expression patterns of clustered *cad20/cad19/cad7 *genes are regulated by species-specific gene regulatory elements in avian and rodent embryos, which is in contrast to the highly conserved expression patterns of HD protein genes in the central nervous system.

## Conclusion

The present study demonstrated that cell populations that express *cad7 *and *cad20 *are diversified in the rat and chick. The expression of these cadherin subtypes demarcates compartments, boundaries, progenitor domains, specific nuclei and circuits during mammalian hindbrain development.

## Methods

### Animals

Animal experiments were carried out in accordance with National Institute of Health guidelines for care and use of laboratory animals. The Committee for Animal Experiments of Tohoku University Graduate School of Medicine approved the experimental procedures described in this study. The midday of the vaginal plug was designated as embryonic day 0.5 (E0.5). Pregnant Sprague-Dawley (SD) rats were purchased from Charles River Japan. *Pax6 *homozygous mutant rat embryos were obtained by crossing of male and female *Small eye *rat heterozygotes (*rSey*^2^/*+*) [52], which were maintained at Tohoku University Graduate School of Medicine.

### Identification of rat *cad7 *and *cad20*

Rat *cad7 *and *cad20 *genomic sequences were identified through BLAST genome search of NCBI and BLAT database at UCSC Genome Bioinformatics. cDNA fragments encoding ORF of rat *cad7 *and *cad20 *were amplified by RT-PCR using oligonucleotide primers designed based on the identified rat genomic sequences. Total RNA taken from the head including the hindbrain of E12.5 rat embryos was purified with RNeasy column (Qiagen, Hilden, Germany), and cDNA was synthesized using oligo dT primer and reverse transcriptase (Superscript II, Invitrogen, San Diego, CA). The primer sets were as follow: 5' fragment of *cad7 *(1–1467: 5'-ATGAAGCTGGGCAAAGTGGAG-3' and 5'-AGTGGTTTCATACTCCATGGC-3'), 3' fragment of *cad7 *(1447–2361: 5'-GCCATGGACTATGAAACCACT-3' and 5'-AGGCTATGAGTACAAACTCTC-3'), 5' fragment of *cad20 *(1–1071: 5'-ATGTGGACTACAGGTAGAATG-3' and 5'-ATTGGATCCTTCCACCTTCAG-3'), and 3' fragment of *cad20 *(1051–2450: 5'-CTGAAGGTGGAAGGATCCAAT-3' and 5'-TGAGAACGTCTGGATTTGGGT-3'). Amplification was performed using a thermal cycler (Mastercycler gradient; Eppendorf, Hamburg, Germany) using *Taq *DNA polymerase (Promega, Madison, WI) under the following conditions: denaturation for 5 min at 96°C, annealing for 1 min at 63.5°C (*cad7*), 60.8°C (*cad20*), extension for 1 min at 72°C, 35 cycles. The amplified products were blunted using T4 DNA polymerase (Invitrogen) and inserted into *Eco*R V site of pBluescript SKII (-). DDBJ accession numbers are AB121031 (rat *cad7*) and AB121033 (rat *cad20*).

### Alignments and phylogenetic analysis of cadherin family

Multiple alignment of amino acid sequences of Cad7 and Cad20 and phylogenetic analysis for full length of amino acid sequences of type II classic cadherins were performed by Clustal W program and gene database. The gene accession numbers of cadherins used to characterize protein sequences are AB121032 (rat *cad19*), AJ007607 (human *cad19*), X95600 (mouse *cad8*), D21253 (mouse *cad11*), D252990 (rat *K-cad/cad6*), D82029 (mouse *cad6*), D42149 (chick *cad6b*), AB035301 (human *cad7*), AK034096 (mouse *cad7*), D42150 (chick *cad7*), AF217289 (human *cad20*), AF007116 (mouse *cad20*), AF459439 (chick *MN-cad*, the same sequence is identified as the chick F-cadherin homolog, AF465257), X85330 (*Xenopus F-cad*), L33477 (human *cad12/Br-cad*), XP_226899 (rat *cad18*), XP_001054792 (rat *cad24/EY-cad*), AY260900 (human *cad24*) and NM_019161 (rat *cad22*). Phylogenetic tree was drawn by Tree View software (Taxonomy and Systematics at Glasgow). The scale bar was set to represent 10% differences.

### Cloning of rat cDNAs encoding transcription factors

To obtain rat *Gsh1*, *Krox20 *and *Gbx2 *cDNAs, the corresponding genomic sequence was amplified by genomic PCR using oligonucleotide primers. Rat *Dbx2 *and *Lbx1 *cDNAs were amplified by RT-PCR using mRNA prepared from E12.5 SD rat embryos. Primer sets were designed based on the sequences, which are homologous to mouse *Gsh1*, rat *Krox20*, rat *Gbx2*, mouse *Dbx2 *and mouse *Lbx1 *mRNAs. Used oligonucleotide primers were as follow, *Gsh1 *(5'-CAGCAGCAGCCAAGGTGATT-3' and 5'-CCACGGAGATGCAGTGAAAC-3'), *Krox20 *(5'-TCAACATTGACATGACCGGAG-3' and 5'-GAATGAGACCTGGGTCCATAG-3'), *Gbx2 *(5'-ACGAGTCAAAGGTGGAAGATG-3' and 5'-TGACTTCGAATAGCGAACCTG-3'), *Dbx2 *(5'-TGCTGACCCAGGACTCAAATT-3' and 5'-GGATACCAAAGAAGCCAGAAG-3') and *Lbx1 *(5'-GAGATGACTTCCAAGGAGGAC-3' and 5'-ATCAGGCTGTAGTGGAAGGAA-3'). Amplification was performed under following conditions: denaturation for 5 minutes at 96°C, annealing for 1 min at 68.1°C (*Gsh1*), 55.5°C (*Krox20*), 60.8°C (*Gbx2*), 60.8°C (*Dbx2*) and 60.8°C (*Lbx1*), extension for 1 min at 72°C, 35 cycles. To clone the amplified products, these fragments were blunted using T4 DNA polymerase (Invitrogen) and inserted into *Eco*R V site of pBluescript SKII (-) (Stratagene, La Jolla, CA). Cloned cDNAs were confirmed by sequencing. DDBJ accession numbers are AB197922 (rat *Gsh1*), AB264614 (rat *Krox20*), AB266843 (rat *Gbx2*), AB121147 (rat *Dbx2*) and AB197923 (rat *Lbx1*).

### Whole-mount *in situ *hybridization

Whole-mount *in situ *hybridization was performed as described previously [27,52]. Dissected embryos were fixed in 4% paraformaldehyde (PFA)/phosphate buffered saline (PBS) overnight at 4°C. Digoxigenin (DIG)-labelled riboprobes were synthesized by *in vitro *transcription with DIG RNA labelling mix (Roche, Mannheim, Germany) and T3 or T7 RNA polymerase (Promega). All synthesized probes were purified with Quick Spin Columns G-25 (RNA) (Roche) to remove unincorporated nucleotides. For detection of *cad7 *mRNA, three kinds of probes transcribed from different *cad7 *cDNA fragments (1–460, 567–1460 and 1992–2361) were used in hybridization. To detect *cad20 *mRNA, four kinds of probes transcribed from different *cad20 *cDNA fragments (1–546, 1051–1497, 1639–1956 and 2014–2403) were mixed in hybridization. Two-colour whole-mount *in situ *hybridization was performed using the protocol described on the internet . For double colour detection, fluorescein-labelled riboprobes for *Dbx2*, *Gsh1*, *Otx2 *and *Gbx2 *were generated with fluorescein RNA labelling mix (Roche), and simultaneously hybridized with DIG-labelled *cad7 *or *cad20 *riboprobes, respectively. After the first colour reaction with alkaline phosphatase (AP)-conjugated anti-DIG antibody (1:5000, Roche) and NBT/BCIP (Wako Pure Chemical Industries, Osaka, Japan), embryos were fixed in 4% PFA/PBS at room temperature overnight and incubated in TBST containing 0.1% Tween20 at 65°C for 1 hour to inactivate AP completely. The second colour signal was detected with AP-conjugated anti-fluorescein antibody (1:5000, Roche) and INT/BCIP (Roche). Drs. I. Matsuo, A. Mansouri and P. Gruss kindly provided mouse *Otx2*, *Six3 *and *Irx3 *cDNAs for synthesis of riboprobes, respectively. Images were recorded by cooled colour CCD camera (Penguin 600CL, Pixera, San Jose, CA).

### *In situ *hybridization

*In situ *hybridization using frozen sections of embryonic tissues was performed as described previously [43]. E12.5 whole rat embryos and heads of E14.5 and E18.5 embryos were fixed in 4% PFA/PBS overnight at °C. For fixation of E20.5 rat foetuses, the brains were dissected and fixed without the pia mater in 4% PFA/PBS overnight. Embryos and foetuses were embedded with optimal cutting temperature (OCT) compound (Sakura, Tokyo) and cut into 12 μm sections with a cryostat (Leica, Nussloch, Germany). DIG-labelled riboprobes were synthesized with T3 or T7 (Promega) or SP6 (Takara Shuzo, Ohtsu, Japan) RNA polymerase and hybridized to sections. To detect the expression of *cad7 *mRNA, two kinds of riboprobes of *cad7 *were generated from different cDNA fragments (1–1467 and 1447–2361), and mixed in hybridization. Riboprobes of *cad20 *were synthesized by from two cDNA fragments (1–1071 and 1051–2450), and used simultaneously in hybridization. Mouse *Gata2*, rat *cad6*/*K-cad *[82] and rat *Sox10 *cDNAs were kindly provided by Drs. M. Yamamoto, M. Tanaka and M. Wegner, respectively. For *in situ *hybridization of the adult brain, 8-week-old male adult rats (postnatal day 60) were deeply anesthetized and decapitated. Brains were immediately dissected out and frozen on powder dry ice. The brains were cut into 14 μm sections, and were post-fixed in 4% PFA/PBS for 15 minutes at room temperature. Sections were acetylated with tri-ethanoamine, HCl and acetic acid for 10 min. The following strategies of hybridization and staining were performed according to the protocol described for embryonic tissues except for changing the concentration of AP-conjugated anti-DIG antibody (1:2000). Images were recorded with colour CCD camera (HC-25000 3CCD, Fuji, Tokyo).

### Immunohistochemistry

Immunohistochemistry using frozen sections was performed as described previously [52]. Embryos and foetuses were fixed as described above for *in situ *hybridization. Antigen-enhanced sections, which were boiled in 0.1 M citric acid solution in a microwave, were incubated with anti-Pax6 rabbit polyclonal antibody [62] (1:500) and anti-Islet1/2 mouse monoclonal antibody (D.S.H.B, 40.2D6, 1:100), which were diluted with 2% goat serum/TBST overnight at 4°C. As secondary antibodies, biotin-conjugated affinity purified anti-rabbit IgG (dilution, 1:200, Jackson Immunoresearch Laboratories, West Grove, PA) and anti-mouse IgG donkey antibodies (1:200, Chemicon International, Inc., Temecula, CA) were used. Signal was enhanced by combination of ABC kit (Vector Laboratories, Burlingame, CA) and enhanced DAB kit (Pierce, Rockford, IL). After *in situ *hybridization for *Sox10 *and *cad7*, sections were incubated with anti-neuron-specific class III β-tubulin antibody (1:2000, Tuj1, MMS-435P, Covance, Madison, WI), and the signal was detected by using Cy3-conjugated affinity purified anti-mouse IgG donkey antibody (1:400, Jackson Immunoresearch Laboratories). Images were recorded with AxioPlanII and AxioCamMRm (Carl Zeiss, Jena, Germany).

### BrdU labelling using whole embryo cultures

Short pulse labelling of bromodeoxyuridine (BrdU, Sigma Chemical Co., St. Louis, MO) for cultured rat embryos was performed as described previously [43]. E12.5 rat embryos were precultured for 1 hour and BrdU solution was directly added to the culture medium. Embryos were exposed to BrdU for 20 min and fixed in 4% PFA/PBS. After detection of *Gata2 *mRNA by *in situ *hybridization, sections were treated with 2N HCl solution for 15 min at 37°C and neutralized in TBST. Sections were incubated with anti-BrdU mouse monoclonal antibody (Becton-Dickinson, Mountain View, CA, 1:50), and the signal was detected with ABC kit and enhanced DAB kit.

## Authors' contributions

MT designed and carried out all experiments in Osumi lab. MT and NO discussed the results and wrote the manuscript together.
